# Decreasing Unexpected Returns to Orthopedic Hand Clinic: Improving Efficiency of Health Care Delivery

**DOI:** 10.1097/pq9.0000000000000107

**Published:** 2018-09-24

**Authors:** Kevin J. Little, Samir Trehan, Roger Cornwall, Stephanie Garrison, Emily Dastillung, Lisa McFadden

**Affiliations:** From the * Department of Surgery, Division of Pediatric Orthopaedics, Cincinnati Children’s Hospital Medical Center, Cincinnati, Ohio; † Department of Orthopaedic Surgery, University of Cincinnati College of Medicine, Cincinnati, Ohio; ‡ Department of Orthopaedic Surgery, Hospital for Special Surgery, New York, N.Y.

## Abstract

**Purpose:**

An unexpected return to clinic (URTC) visit can place a substantial financial burden on patients and families while stressing the health care system. Our SMART aim was to decrease the rate of URTC visits from 1.8 per 100 patient follow-up visits by 50% using quality improvement methodology.

**Methods:**

The rate of URTC visits was tracked at our tertiary care pediatric hospital from February 1, 2014, to May 31, 2015, using a weekly P-chart. Interventions were studied from January 1 to May 31, 2015. Pareto charts determined the common causes of URTC visits. Interventions were studied using Plan-Do-Study-Act cycles. Medical charges for URTC patient visits were collected and patients/families were given a cost survey to determine nonmedical costs associated with the clinic visits.

**Results:**

Cast issues (50.5%) were most common, followed by new symptom/complaints (29.5%), and persistent or worse symptoms (15.2%). Following interventions, URTC rates decreased from 1.8 to 0.7 (⇓62%) per 100 follow-up visits during the study period. Interventions were targeted toward cast use and improved patient education via standardized materials. The average URTC resulted in $350.38 of charges. Additionally, the average URTC cost families $70 for a half day of lost wages and travel expenses.

**Discussion:**

Applying quality improvement methodology to URTC visits by standardizing patient education and minimizing cast usage resulted in a substantial decrease in the number of patients returning to clinic, both for scheduled follow-ups and unexpectedly. This improvement resulted in a savings of more than $420 per visit saved, including medical and nonmedical costs.

## INTRODUCTION

Over the last several years, a major focus of the health care industry has been to track the quality, safety, and value of medical care given by the health care system.^1^ Quality improvement (QI) methodology is a formalized approach to analyze the performance of a health care delivery system, and to assess the impact and results of changes made to the system. A QI program involves systematic activities that are organized and implemented by a health care provider to monitor, assess, and improve the quality of health care being delivered. There are many models of quality improvement utilized in health care delivery, and our hospital adopted the System of Profound Knowledge, popularized by W. Edwards Deming.^2,3^ This model involves the interrelationship of 4 main domains of quality improvement: the theory of knowledge, psychology, understanding variation, and the appreciation for a system. By utilizing this model, the system of health care delivery can be improved to maximize patient outcomes.

In our Pediatric Orthopaedic department, a small, but persistent, number of patients were calling the office to arrange urgent and unexpected follow-up visits. Facilitating these urgent clinic visits placed substantial strains and inconvenience on the medical system, medical provider, patient, and/or family. It has been reported that surgical follow-up care can be expensive in terms of medical and nonmedical costs, which can disproportionately impact low-income patients.^4^

We hypothesized that we could decrease unexpected return to clinic (URTC) visits by increasing health care efficiency and safety via a Deming model of QI methodology. Specifically, we collected data to identify indications for URTC visits. We noted that many URTC visits could be prevented with improved patient education and avoidance of casting. We then designed and implemented interventions to address these issues and monitored the effect on URTC visits and cost.

## MATERIALS AND METHODS

Institutional review board exemption was provided by our institution for QI projects.

We defined an URTC visit as an unscheduled visit that occurred 30 days before the next scheduled visit, or within 30 days of a previous visit in which the patient was discharged from clinical care. URTC visits are reported as the number of unplanned visits per 100 patient follow-up visits. URTC visits were tracked in the Pediatric Orthopaedic department from February 2014 through December 2014 using weekly and monthly P-charts (control charts that track the rate over time). Baseline data were analyzed from 2014 to determine leading indications for URTC visits and identify potential areas of intervention.

**Fig. 1.**
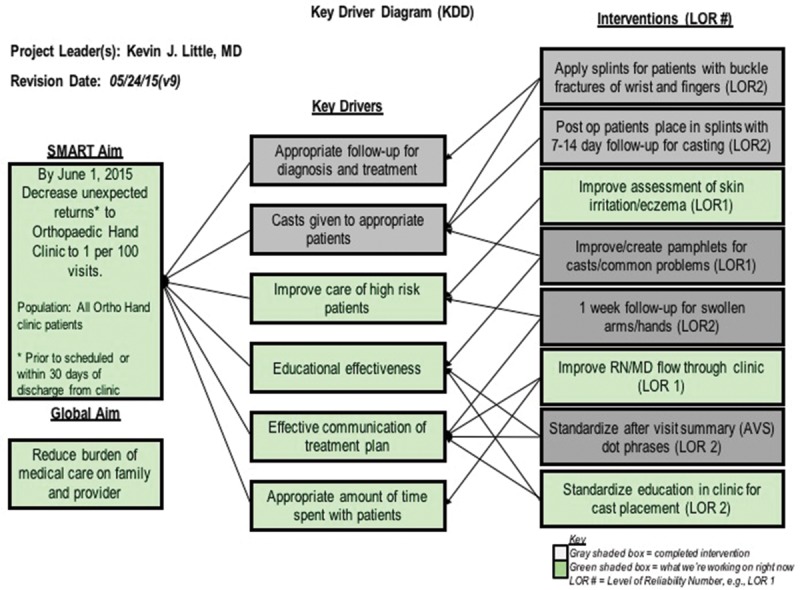
The Key Driver Diagram used to identify key issues to address to decrease the rate of unplanned returns to clinic.

**Fig. 2.**
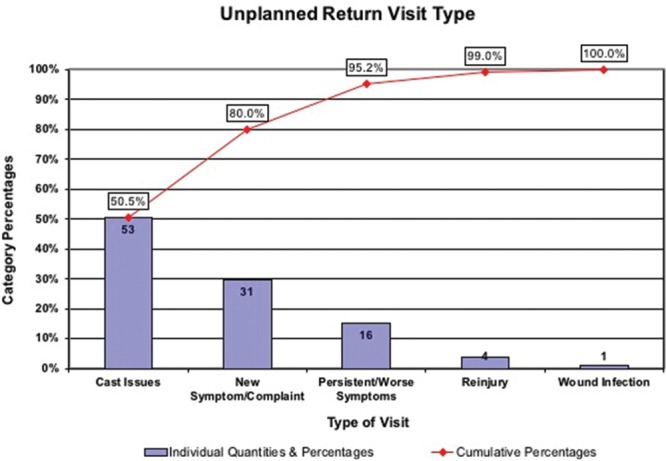
A Pareto chart of baseline data that demonstrates the most common reasons for unplanned returns to clinic.

From January 1, 2015, through May 31, 2015, QI strategies were implemented to decrease the rate of URTC visits. Our specific, measurable, actionable, relevant, time-bound (SMART) aim was to decrease the rate of URTC visits to the Orthopaedic Hand and Upper Extremity clinic by 50% by May 31, 2015. A process flow chart was created to map the scheduling of Orthopaedic clinic visits and Pareto charts were utilized to stratify data into specific URTC visit categories. A Key Driver Diagram (Fig. 1) was created to identify the critical categories that could most affect URTC visit rates and guide intervention design. Specific interventions are discussed in the *Results*. Plan-Do-Study-Act (PDSA) cycles (Fig. 2) were utilized to assess whether the incremental changes made to the system resulted in an improvement.

As a result of cast issues being the primary driver of URTC visits as noted in the Pareto Chart, initial PDSA cycles were tailored around cast issues to drive down the number of cast issues noted. The first PDSA cycle involved replacing casts with removable splints for all distal radius buckle fractures for a single practitioner for 1 week, which was then expanded to the entire Hand team (2 physicians and a physician assistant) and eventually included all buckle fractures of the wrist, hand, and digits. Additionally, postoperative splints were placed instead of casts for patients who did not have exposed pins and were older than 5 because an age histogram demonstrated a break in the data above this age. We also tracked patients who had irritation or reactions to waterproof casting and provided education to providers and patients regarding selection of appropriate cast liners. We noted a 1% rate of patients returning to clinic after splinting for fractures that would have otherwise been casted before our interventions. PDSA cycles were also performed to address the new symptoms/complaints and persistent symptoms categories by standardizing physician and nurse patient education during clinic visits, improving discharge summaries to reflect pertinent and disease-specific instructions, identifying the appropriate timeliness for initial patient scheduling, and creating or improving handouts given to patients for common disease states.

Medical charges associated with each URTC visit was collected for all patients from January 2014 through May 2015. Additionally, URTC visit patients and families were given a brief survey to determine the out-of-pocket nonmedical costs associated with these visits, such as lost work time, travel expenses, child care expenses, and time away from school.

Balancing measures, including unexpected emergency room (ER) visits, were tracked during the study period as well.

### Statistical Analysis

Statistically significant differences in the rate of URTC visits were determined by a change in the centerline of the P-chart, which requires 8 consecutive points below the previous centerline (equivalent to a 99.7% chance that this change is not due to random variation, or *P* < 0.003).

## RESULTS

**Fig. 3.**
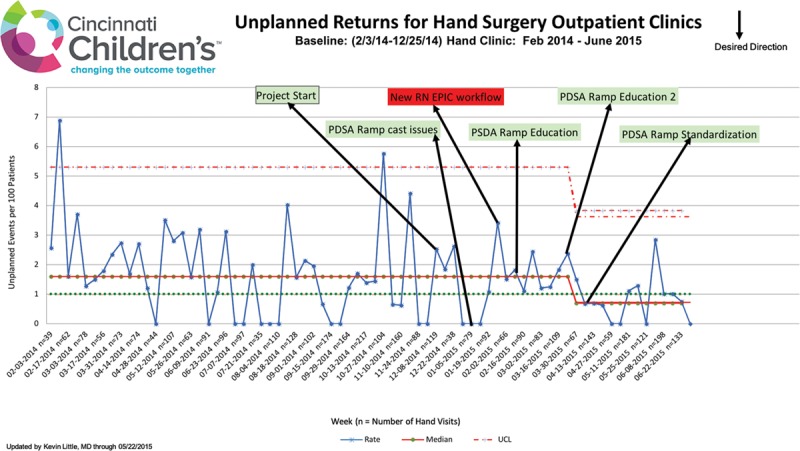
P-chart demonstrating the weekly rate of URTC visits during the study period.

During the baseline period from January 2014 through December 2014, the rate of URTC visits was 1.8 per 100 follow-up visits. Pareto charts (Fig. 3) demonstrated that the majority of URTC visits were related to cast issues (51%), followed by new symptom/complaints (29%), persistent/worse symptoms (15%), reinjury (4%), and wound infection (1%). Casts were most frequently too loose (39%), fell apart (19%), were removed by the patient (19%), got wet if they were not waterproof (12%), were constricting (11%), or had a foul odor (2%). Cast issues more commonly happened postoperatively (5% of surgical patients), rather than in nonsurgical casted patients (1.5%). Additionally, 71.4% of cast issues happened in the first 2 weeks of initial placement. There was additionally no change in the rate of institutionally tracked ER visits for cast-related or other orthopedic issues over the course of the study.

**Fig. 4.**
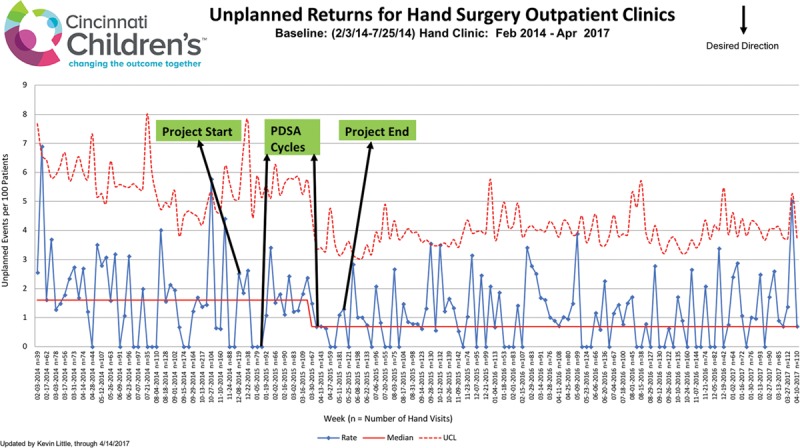
P-chart demonstrated that the weekly rate of unexpected returns to clinic visits were sustained for 1 year following completion of the project.

Over the course of the study period from January 1 to May 31, 2015, the rate of URTC visits decreased from 1.8 per 100 visits to 0.7 per 100 visits (62%), reflected by a new centerline on the p-chart (*P* < 0.003; Fig. 1). URTC rates were tracked for 1 year following completion of the project and were unchanged, indicating a sustained effect of the interventions that were initiated (Fig. 4). Each URTC visit averaged $350 of medical charges ($47.14 in professional fees, $303.24 in hospital fees, radiology fees, and supplies) and $70 of nonmedical out-of-pocket costs ($40 for half day lost work and $30 for 50 miles of round-trip travel at $0.60 per mile). Charges for professional fees were low because most patients returned during the 90-day global billing period for fracture or operative care. In addition, 60% of survey respondents had to arrange childcare for other children (typically through family or friends at no additional cost) and patients averaged a half day of lost school time to attend these appointments.

## DISCUSSION

URTC visits to the orthopedic clinic can cause substantial economic, social, and time constraints on the medical system, provider, family, and patient. Reducing the need for follow-up appointments, whether planned or unplanned, streamlines the delivery of orthopedic care and maximizes the value that orthopedic surgeons can provide for their patients, while simultaneously minimizing the costs associated with that care. The transition from fee-for-service payor models to Accountable Care Organizations or value-based care models will only serve to drive this change as hospitals and physicians attempt to minimize expensive care that provides marginal benefit to patients. QI methodology, as demonstrated in this study, is a useful tool to decipher what aspects of the medical care delivery system can be targeted to improve healthcare efficiency and value.

Previous studies have looked at the rates of unplanned readmissions to the hospital following hand and upper extremity surgery (0.78%),^5^ plastic surgery (2.40%),^6^ and neurosurgery (11.2%)^7^ in children. These studies identified that postoperative infections^5,6^ were the most common reason for readmission and demonstrated that medical comorbidities,^5–7^ increased duration of operative procedures,^6,7^ and length of hospitalization^6,7^ following surgery were predictive of unplanned readmissions. Additional studies have reviewed unplanned visits to the emergency department or urgent care following pediatric urologic surgery and noted a rate of 2.54%, with the most common causes being postoperative pain and infection.^8^ Although these were the first studies to look at unplanned readmission to the hospital system following pediatric surgery, none of them focused on measures to reduce these rates. Shermont et al.^9^ focused on standardization of discharge bundles combined with teach back methodologies to improve the rate of 7-day and 30-day readmissions following discharge from a pediatric hospital. They used PDSA cycles to implement a standardized discharge plan including medication reconciliation, scheduled follow-up appointments, and verifying that the patient/family understood the discharge plan and a number to call for any questions or concerns. By gradually implementing this over 16 inpatient units, they were able to decrease readmissions by 8% at 7 days and 10% at 30 days. Similar to our study, in the study by Shermont et al.,^9^ multiple small tests of change were used to gradually implement their improvement strategy and obtain a significant decrease in their outcomes measure.

To our knowledge, this study is the first to evaluate unexpected returns to the outpatient clinic following outpatient treatment or surgical treatment for pediatric upper extremity injuries or ailments, with noted rates similar to those published for pediatric upper extremity and plastic surgery. Using Pareto charts, we were able to accurately identify the most common causes of these unexpected returns and begin planning PDSA cycles to affect changes to these causes. Most unplanned return visits were due to cast issues, many of which were preventable or avoidable. Careful patient selection is critical to the success of casting. Our institution provides waterproof casts to appropriate patients with minimal swelling and no open wounds. However, children with a propensity for eczema have a higher likelihood of irritation/skin reaction to the waterproof liner, and we now routinely ask about eczema or skin conditions in patients who choose waterproof casts. Additionally, patients with significant swelling are at high risk of cast looseness once the swelling subsides, and may benefit from additional splinting or routine cast changes. By facilitating appropriate timing of the initial clinic visit 5−7 days following injury, the peak swelling has subsided and casts are more safely placed without as much risk of subsequent loosening or displacement. We also noted a higher rate of cast issues in postoperative patients, and now routinely place splints instead of casts for patients of appropriate age and surgery type. Many of these patients return in 10−14 days for a wound check and transition to a waterproof cast, although casts are placed in patients with a substantial travel time or added expense to avoid additional clinic visits when possible. We did not place splints on patients with exposed pins, or in children younger than 5, in whom a change to a waterproof cast may induce anxiety, pain, and difficulty with cast placement in the early postoperative period. This practice change did not significantly alter the number of patients returning to clinic in the postoperative period, as many patients would initially have a postoperative cast placed in the operating room and return in 10−14 days for a waterproof cast, especially during the summertime.

An additional component to prevent URTC visits is to provide appropriate patient education regarding their condition. We were able to standardize the education given to patients for many common disease states by utilizing templates in the patients’ discharge summaries to highlight what the patient should expect over the course of their treatment, provide appropriate teaching regarding when to notify the office should problems arise, and have a physical document that the patient can take home and read at their leisure to gain more insight into their disease and treatment. We additionally implemented standardized discharge summaries and teaching for patients in clinic, similar to the QI methodologies used by Shermont et al.^9^

This intervention reinforces the verbal communication given by the physician, physician assistant, nurse, or orthopedic technologist, which is often difficult to remember in a short clinic visit.^10^ Additionally, casted patients and patients with common disease states such as nail bed injuries, trigger thumbs, and ligament sprains or avulsion fractures were given pamphlets regarding their diagnosis, treatment, and expected outcomes to help with their education and knowledge retention following their initial clinic visits.

The costs associated with URTC visits were tracked during the study period. Professional fees resulted in approximately $50 in medical charges per visit, while hospital fees were 6-fold more expensive. The professional fees were lower, because most URTC visits were during the 90-day global period for fracture care or surgery, where professional fees are not charged. However, charges for cast supplies, repeat radiographs indicated for patients with pain or swelling as their presenting complaint, or facility fees were the biggest driver of medical charges for these visits. Although many of these charges are covered by medical insurance companies, deductibles may be charged to the patient on top of out-of-pocket nonmedical expenses that can impose a substantial financial burden on patients.^4^ Most of the these nonmedical costs were related to transportation and lost time from work. Although these are not exclusive to URTC visits, many families will choose routine follow-up visits to coordinate care with days off of work, or for other errands or appointments to decreases the out-of-pocket costs associated with their care. Thus, routine follow-up appointments, made at the patient’s and families convenience, present less of a cost burden than unexpected visits, which can result in making appointments at locations that aren’t as close to the families home or during normal work or school hours. Calculating hospital charges is not a true representation of the cost of care, which is much more difficult to calculate, especially because each individual URTC visit required different elements of care. However, by eliminating URTC visits, the charges associated with supplies, equipment, and salary for those practitioners involved in each patient care episode will be reduced, with overall savings to the health care industry without sacrificing the quality of care.

Applying QI methodology to minimize URTC visits in our Pediatric Orthopaedic Hand and Upper Extremity clinic by improving/standardizing patient education and restricting cast usage resulted in a significant decrease in the URTC visit rate. This improvement resulted in savings of $420 per visit saved, including medical charges and nonmedical out-of-pocket costs borne by the families. Overall improvements in health care efficiency and value will be rewarded as medical care is transitioned away from fee-for-service toward value-based care models.

## DISCLOSURE

The authors have no financial interest to declare in relation to the content of this article.

## References

[1] Brighton BK National Surgical Quality Improvement Program-Pediatric (NSQIP) and the Quality of Surgical Care in Pediatric Orthopaedics. J Pediatr Orthop. 2015;35:S48 S50. 26049305 10.1097/BPO.0000000000000548

[2] Lynn ML Osborn DP Deming’s quality principles: a health care application. Hosp Health Serv Adm. 1991;36:111 120. 10108969

[3] Duncan RP Fleming EC Gallati TG Implementing a continuous quality improvement program in a community hospital. QRB Qual Rev Bull. 1991;17:106 112. 1852431 10.1016/s0097-5990(16)30437-7

[4] Scott AR Rush AJ 3rd Naik AD Surgical follow-up costs disproportionately impact low-income patients. J Surg Res. 2015;199:32 38. 26013443 10.1016/j.jss.2015.04.013

[5] Thibaudeau S Anari JB Carducci N 30-Day readmission after pediatric upper extremity surgery: analysis of the NSQIP database. J Pediatr Surg. 2016;51:1370 1374. 27199258 10.1016/j.jpedsurg.2016.04.012

[6] Tahiri Y Fischer JP Wink JD Analysis of risk factors associated with 30-day readmissions following pediatric plastic surgery: a review of 5376 procedures. Plast Reconstr Surg. 2015;135:521 529. 25357160 10.1097/PRS.0000000000000889

[7] Sherrod BA Johnston JM Rocque BG Risk factors for unplanned readmission within 30 days after pediatric neurosurgery: a nationwide analysis of 9799 procedures from the American College of Surgeons National Surgical Quality Improvement Program. J Neurosurg Pediatr. 2016;18:350 362. 27184348 10.3171/2016.2.PEDS15604 PMC5445382

[8] Arlen AM Merriman LS Heiss KF Emergency room visits and readmissions after pediatric urologic surgery. J Pediatr Urol. 2014;10:712 716. 24239305 10.1016/j.jpurol.2013.09.028

[9] Shermont H Pignataro S Humphrey K Reducing pediatric readmissions: using a discharge bundle combined with teach-back methodology. J Nurs Care Qual. 2016;31:224 232. 26845419 10.1097/NCQ.0000000000000176

[10] Kessels RP Patients’ memory for medical information. J R Soc Med. 2003;96:219 222. 12724430 10.1258/jrsm.96.5.219 PMC539473

